# (Ultra) High Pressure Homogenization for Continuous High Pressure Sterilization of Pumpable Foods – A Review

**DOI:** 10.3389/fnut.2014.00015

**Published:** 2014-08-19

**Authors:** Erika Georget, Brittany Miller, Michael Callanan, Volker Heinz, Alexander Mathys

**Affiliations:** ^1^German Institute of Food Technologies (DIL), Quakenbrueck, Germany; ^2^Institute of Food Chemistry, Leibniz University of Hanover, Hanover, Germany; ^3^Nestlé Research Center, Lausanne, Switzerland

**Keywords:** ultra high pressure homogenization, bacterial spore, sterilization, food, continuous processing

## Abstract

Bacterial spores have a strong resistance to both chemical and physical hurdles and create a risk for the food industry, which has been tackled by applying high thermal intensity treatments to sterilize food. These strong thermal treatments lead to a reduction of the organoleptic and nutritional properties of food and alternatives are actively searched for. Innovative hurdles offer an alternative to inactivate bacterial spores. In particular, recent technological developments have enabled a new generation of high pressure homogenizer working at pressures up to 400 MPa and thus, opening new opportunities for high pressure sterilization of foods. In this short review, we summarize the work conducted on (ultra) high pressure homogenization (U)HPH to inactivate endospores in model and food systems. Specific attention is given to process parameters (pressure, inlet, and valve temperatures). This review gathers the current state of the art and underlines the potential of UHPH sterilization of pumpable foods while highlighting the needs for future work.

## Introduction

Mechanical homogenization was defined as the capability of producing a homogeneous size distribution of particles suspended in a liquid, by forcing the liquid under the effect of high pressure through a disruption valve (1). The first occurrence of homogenization for the stabilization of food and dairy emulsions is dated from 1900 at the Paris World’s Fair and was first patented in 1899 by Auguste Gaulin as an invention for “intimately mixing milk” using pressures up to 30 MPa (French Patent no. 295.596) (2). Since then conventional homogenization extended the pressure range until 50 MPa. High pressure homogenization (HPH), also known as dynamic HPH, has been repeatedly highlighted for its potential for pasteurization of food matrices at reduced thermal loads (3–6). HPH enables pressures 10–15 times higher than traditional homogenizers and covers the range 100–400 MPa within which the upper pressure range from 300 to 400 MPa has been referred to as ultra high pressure homogenization (UHPH) (7). In this review, the range 100–200 MPa will be referred to as HPH and the range >200 MPa as UHPH. Inactivation of vegetative microorganisms has been demonstrated (3) and is achieved through a combined action of cavitation, shear stress, turbulence, impingement, and high pressure leading to disruption of the vegetative microorganism (8). It was shown that the efficiency of UHPH for vegetative microorganisms inactivation increased with the pressure level, the number of passes, the inlet, and valve temperatures but the exact mechanisms and interaction between parameters remain, however, to be elucidated (7). The valve temperature (*T*_valve_) showed a variable contribution to inactivation. While for *T*_valve_ <60°C, the mechanism of inactivation suggested a synergetic action of all stress factors, *T*_valve_ >80°C seemed to lead to a temperature driven inactivation (7).

The progression toward UHPH has also opened the door to new sterilization opportunities, which might go past the initial theory that it might not be possible to inactivate spores by HPH (3). Bacterial spores, whose resistance to lethal treatments intensify the concern of their threat to food microbial safety (9), and their behavior when processed through UHPH are of prime interest. Comparatively, promising results could be achieved using high isostatic pressure thermal sterilization in model systems or products such as baby food (10, 11). Synergetic effects of temperature and isostatic pressure were demonstrated enabling a reduction of the total thermal load. However, this process is, to date, not available at industrial scale, and remains a batch process, which leads to significantly higher production costs than conventional thermal processes. Moreover, the minimum pressure required to achieve spore inactivation at high temperature is significantly higher than the UHPH pressure range (>500 MPa) (10, 11). The existing literature focusing on bacterial spore inactivation by UHPH is more recent and still faces inhomogeneity in conclusions. A recent extensive review of the technological aspects and potential applications of UHPH is available for the reader but only succinctly touched upon the topic of inactivation of bacterial spores by UHPH (7). In this short review, the existing literature focusing on bacterial spore inactivation by HPH and UHPH in different matrices, as well as the main learnings reached in terms of sterilization potential, is reviewed. A particular attention is given to the individual process parameters and target organisms in correlation with the achieved inactivation.

## Bacterial Spore Inactivation by HPH/UHPH in Model Systems and Food Matrices

Detailed work has been reported on the impact of high pressure on bacterial spores (10, 12, 13). It could be shown that high pressure (>600 MPa) and temperature (>60°C) have a synergistic impact on bacterial spore inactivation (14). High pressure germination of bacterial spores was also achieved in the range 150–300 MPa/30–55°C (15, 16), corresponding to ranges achieved by HPH/UHPH. Yet, it was shown that very short exposure to high pressure might not be sufficient to trigger germination if immediately followed by atmospheric pressure as was shown for *Bacillus subtilis* spores treated at 150 MPa/37°C for a few seconds (17). Furthermore, several investigations reported with HPH/UHPH and spores failed at showing significant inactivation potential (4). However, a more in depth analysis of the work conducted this far on bacterial spore inactivation by HPH/UHPH shows that, often, a suboptimal process window might be responsible for this result.

The first study on the impact of HPH on bacterial spores was reported by Feijoo et al. (18). While the inlet and outlet temperatures were given, the actual maximal temperature of processing was not stated. A maximum spore reduction of 68% – that is 0.55 log_10_ – was reported for 200 MPa and 50°C inlet temperature and though not successful in full spore inactivation, this work opened a new field of investigation.

Several following investigations within the HPH or UHPH domain also reported failure to strongly inactivate bacterial spores of different genera, species, and strains in model systems or food matrices (19–24) (Table 1). A common point to all these investigations is that the maximum valve temperature achieved was not stated, and/or relatively low, when one considers the applied inlet temperatures and estimates the valve temperature on the basis of ~20°C increase per 100 MPa (3). While some authors attributed spore resistance to a lower exposure area of proteins in the spore, as well as inner structure cross protection by Dipicolinic acid (DPA) (19), one might rather suspect that the thermal load necessary to achieve spore inactivation was not achieved and/or that the contribution of the other stress factors was insufficient to trigger sufficient inactivation.

**Table 1:** **Overview of literature on non-successful HPH/UHPH inactivation of bacterial spores**.

Equipment	Matrix	Spore strain	Initial count (spore/mL)	Maximal reduction [log_10_ (N/N_0_)]	Pressure (MPa)	*T*_inlet_ (°C)	Max *T*_valve_ (°C)	Source
Microfluidizer^®^	Ice cream	*B. licheniformis* ATCC 14580	2.00E + 04	0.55	200	50	?	(18)
Niro Soavi homogenizer	Double distilled water	*B. cereus* SV3, SV98, *B. subtilis* SV50, SV108	1.00E + 07 – 1.00E + 08	<0.5 with single pass – five with three cycles	150	20	?	(21)
Panda – Niro Soavi	Laboratory medium at pH 4.5 and 3.5	*A. acidoterrestris* DSMZ 2498, Γ4, and c8	1.00E + 05	0.67 (140–170 MPa)	140–170	?	?	(19)
Panda – Niro Soavi	Malt extract broth (pH 4.5) and apple juice (pH 3.7)	*A. acidoterrestris* DSMZ 2498 and Γ4	1.00E + 05	0.82 ± 0.07	140	?	?	(23)
SFP FPG 12500	Broth pH 4	*A. acidoterrestris* N-1100, N-1108, N-1096, SAC, OS-CAJ	1.00E + 06	<0.5	100, 200, 300	?	?	(24)
SFP FPG 7400H:350	Skim milk	*G. stearothermophilus* ATCC 7953, *Clostridium sporogenes* PA 3679	1.00E + 05	0.67 (16 passes – 300 MPa)	100–300	45	84	(22)
SFP FPG 11300	Milk 3.5% fat	Naturally present spores	5.00E + 01	1.1 (200–300 MPa)	100, 200, 300	30, 40	103	(20)

Some authors attempted to combine HPH or UHPH with additional hurdles such as low pH (19, 21), dimethyl dicarbonate 250 ppm (24), or sodium benzoate (23). While Bevilacqua et al. (23) suggested that an interaction between HPH and sodium benzoate (80 mg/L) could occur in some cases (e.g., apple juice) for a specific strain of *Alicyclobacillus acidoterrestris* at low inoculums, this could not be generalized and all in all, it appears that none of these treatments were sufficient to achieve a cumulative or synergetic response in the spore inactivation by HPH or UHPH.

In the work of Chaves-López et al. (21), inactivation improved through multiple cycles and three cycles led to 5 log_10_ reduction of *Bacillus cereus* spores (SV3, SV98, SV50, and SV108) and a corresponding DPA release of up to 52%. This led the authors to suggest that the ultra-rapid depression during HPH treatments might cause a mechanical disruption of the coat and cortex, allowing DPA to leak out. However, it is reasonable to wonder why, with over 99.99% of spores inactivated, the DPA release did not reach 100%. This difference in result suggests that other mechanisms might be involved, leading to spore incapacity to grow post multiple cycles of HPH. Moreover, the mention that during multiple cycles, samples were successively treated without any storage suggests that the new inlet temperature might have been close to the first outlet temperature estimated at 45°C and likely even higher for the third cycle. No mention is made of the valve temperature and it is hard to estimate what might have been the most drastic conditions applied to the spores. An increasing processing valve temperature could be one possible explanation in the increasing inactivation observed. This hypothesis would also support why later work on thermo-resistant spores of *Geobacillus stearothermophilus* ATCC 7953 did not show any inactivation with up to 16 passes at 300 MPa and a maximum valve temperature of 84°C (22). The interest of the work done by Pinho et al. (22) lies in the choice of strains and inoculation level, *G. stearothermophilus* ATCC 7953 and *Clostridium sporogenes* PA 3679 both at 10^5^ spore/mL, the former being the reference strain for wet heat sterilization and thus highly relevant when looking at UHPH sterilization. The study conducted at pressures between 100 and 300 MPa, however, concluded on an absence of inactivation of spores of both strains based on an inlet temperature of 45°C and maximum valve temperature of 84°C. Furthermore, treatment did not change the *D*- and *z*-values of *G. stearothermophilus* and *C. sporogenes*, indicating that UHPH treatment at 300 MPa did not sensitize the spores to thermal treatments nor cause germination. These results contradicted the results of Chaves-López et al. (21) and seem more likely considering the very short exposition time to high pressure (<1 s). The authors did not investigate higher inlet temperatures and therefore, did not reach higher temperature at the valve either. The conclusion on the absence of sterilization potential of UHPH might, here again, be linked to treatments done at too low valve temperatures to lead to inactivation.

Yet, multiple successful attempts at bacterial spore inactivation by HPH/UHPH were also reported (Table 2) (25–33). Most of this recent work was conducted in animal and vegetal milks and with higher pressures and inlet temperature ranges. Although the reported holding time at high temperature varies between studies, it could overall be approximated to <1 s. In spite of this very short time, one must consider the corresponding valve temperature (Table 2). For all studies where a full and durable inactivation of the native or inoculated spore flora could be achieved, the maximum temperature achieved (directly after the valve) was above 130°C and the pressure at 300 MPa. These recent studies are to date the best examples that UHPH is a promising technology to achieve commercial sterility of pumpable foods at the condition of using sufficiently high homogenization pressures and high inlet temperatures. A patent was published on January 26, 2012, which focuses on the use of UHPH for simultaneous sterilization and homogenization of pumpable foods and using the case of soy milk (34). The results in this patent correspond to the ones obtained in the studies introduced here above and confirm the potential of UHPH to inactivate endogenous spore formers. It nonetheless remains that the role of various stress factors during UHPH has not been investigated and the inactivation mechanisms remain to be established. While Valencia-Flores et al. (30) claims that the thermal treatment in combination with physical forces led to inactivation of endogenous vegetative cells and spores, no formal proof is given. The resulting high valve temperature seems to be a pre-requisite to any successful UHPH inactivation of mesophilic spore strains whose thermal resistance is limited. Using the indicated residence time at high temperature as well as the *D*- and *z*-values of isolated strains within the product, it would have been interesting to estimate how much inactivation could be associated to the thermal load only. Additionally, assessing the full inactivation potential via inoculated samples would also have been interesting and was not conducted in the work of Valencia-Flores et al. (30). Also little work was conducted with highly thermo-resistant bacterial strains (22). While the non-inoculated samples sterility could be assessed by shelf-life studies, the absence of high thermostable strains suggests a potential hazard in the process validation. Very recent work by Amador-Espejo et al. (31) initiated exploration of UHPH thermophilic spore inactivation with inoculation of whole UHT milk and showed that with an inlet temperature of 85°C, *G. stearothermophilus* spores could be inactivated. However, the strain used in this work was not ATCC 7953, the official wet heat sterilization indicator. If UHPH proves to be a thermally driven process, *G. stearothermophilus* ATCC 7953 would be recommended as indicator due its high resistance to wet heat inactivation (35).

**Table 2:** **Overview of literature on successful HPH/UHPH inactivation of bacterial spores**.

Equipment	Matrix	Spore strain	Initial count (spore/mL)	Maximal reduction [log_10_ (N/N_0_)]	Pressure (MPa)	*T*_inlet_ (°C)	Max *T*_valve_ (°C)	Source
SFP benchtop homogenizer nG12500	UHT whole milk	*B. cereus* (CECT 5144), *B. licheniformis* (DSMZ 13), *B. sporothermodurans* (DSMZ 10599), *B. coagulans* (DSMZ 2356), *G. stearothermophilus* (CECT 47), *B. subtilis* (CECT 4002)	~1.00E + 06	>5 (for all strains at 300 MPa/85°C)	300	55, 65, 75, 85	139.0 ± 1.3	(31)
SFP FPG 11300	Soy milk	Naturally present spores	2.34E + 02	2.13 (300 MPa)	200, 300	40	108	(25, 26)
SFP FPG 11300	Almond beverage	Naturally present spores (mesophilic and *B. cereus*)	1.00E + 04 – 1.00E + 03 (*B. cereus*)	ND after 30°C/20 days (300 MPa/65–75°C)	200, 300	55, 65, 75	129.3 ± 12.6	(30)
SFP FPG 11300	Almond milk – soy milk	Naturally present spores	1.51E + 03 (S) – 1.62E + 04(A)	ND (200 MPa/75°C – 300 MPa/65–75°C)	200, 300	55, 65, 75	135.7 ± 1.5	(27)
SFP FPG 11300	Almond beverage – soy milk	*Paenibacillus taichungensis*, *B. cereus*, *B. subtilis*, *Lysinibacillus* spp.	1.00E + 05 – 1.00E + 06	ND for all except *B. cereus:* ~5	300	55, 65, 75, 85	138.0 ± 1.4	(28)
SFP FPG 11300	Soy milk	Naturally present spores (mesophilic and *B. cereus*)	2.88E + 03 – 3.55E + 03 (*B. cereus*)	ND after 30°C/20 days (300 MPa/75°C)	200, 300	55, 65, 75	135.7 ± 1.5	(29)
SFP FPG 11300	Milk 3.5% fat	Naturally present spores	1.00E + 01	ND after 30°C/15 and 45°C/7 days (300 MPa/75–85°C)	200, 300	55, 65, 75, 85	139.0 ± 2.7	(32)
SFP FPG 11300	Soy milk	Naturally present spores (mesophilic and *B. cereus*)	1.51E + 02 – 1.95E + 02 (*B. cereus*)	ND after 30°C/20 and 55°C/10 days	300	80	144	(33)

*ND: not detected*.

## Conclusion

Extensive work has been conducted on the impact of high isostatic pressure on bacterial spores and it has been shown that high pressure and temperature have a synergetic impact on bacterial spore inactivation. Significant germination of bacterial spores could also be achieved in the range 150–300 MPa, which would correspond to the range achieved by HPH. However, very short exposure to high isostatic pressure did not permit to trigger germination if immediately followed by atmospheric pressure as is for instance the case in HPH/UHPH processing. This might explain the absence of significant germination by HPH/UHPH reported by some authors because the spores are exposed to high pressure for less than a second. The occurrence of spore germination could be interesting to allow for an easier inactivation by reducing the spore resistance to further thermal hurdles (for instance, a second UHPH cycle or the addition of a holding section at pasteurization temperature post UHPH) and future work will need to validate the (absence of) impact of HPH/UHPH on spore germination.

Most of the work which has been conducted on the investigation of HPH/UHPH impact on bacterial spore inactivation is recent but has managed to raise significant hope and interest in this technology for continuous sterilization of pumpable foods. In light of the results gathered in this short review, the best inactivation could be achieved by combining the highest pressures (≥300 MPa) and high inlet and valve temperatures for short holding time (less than 1 s). One might expect a beneficial effect of UHPH on inactivation of bacterial spores through synergetic effect of pressure, shear, cavitation, temperature, and turbulence but this synergy remains to be established. Although the importance of temperature has been made clear (31), no studies found thus far were able to validly establish the effect of the other stresses such as cavitation or shear in the inactivation. Moreover, an estimation of the expected thermal inactivation through the thermal inactivation kinetic modeling of the strains considered and the residence time could not be found. This could be a simple, yet interesting approach to validate the role of temperature versus other factors.

Additionally, this review underlines the absence of a clear surrogate to validate sterilization by UHPH. Many studies in food matrices looked at *B. cereus* spores and concluded on sterilization with full inactivation of this endogen pathogenic strain in the matrix. However, if the temperature is the main factor influencing inactivation, then validations ought to be conducted with thermostable strains such as the wet heat sterilization indicator *G. stearothermophilus* ATCC 7953 spores. Future studies with this indicator and higher inlet and valve temperature could be useful to validate the UHPH sterilization over a broader range of resistant spore formers.

One further key interest of this technology could lie in the fusion of two energy consuming steps, namely sterilization and homogenization, in one unit operation for classically homogenized fluids such as milks, thus reducing overall processing complexity and costs. For this, however, the physico-chemical and nutritional properties of the finished products will also need to be considered. Some of the studies considered in this review looked at the quality parameters of vegetal milks after UHPH and could show benefit from UHPH at 300 MPa on the color and colloidal stability of soy milks by comparison to UHT (25, 29, 33). It was also found that less furan were produced by UHPH at 300 MPa than by UHT processing (28).

Finally, all the trials listed in this review were conducted using pilot scale equipment and scalability of the UHPH equipment to industrial level is not yet given. GEA Niro Soavi seems, to date, to be close to achieving this goal with the UHP4000 prototype with an aseptic design allowing for SIP and CIP cleanability on the process side and on the aseptic containment system for sterile product processes. This system operates from 100 up to 500 L/h (36, 37). BEE international commercializes homogenizers performing at 310 MPa up to 1500 L/h (38), while the spin-off Ypsicon, founded in 2013, advertises an equipment with aseptic filling applying 350 MPa and 1000 L/h (39). However, to date, no example of the use of these two pieces of equipment for commercial products by industry was found.

In conclusion, the state of the art given in this review suggests that UHPH has indeed strong potential as an emerging technology for sterilization of pumpable foods in combination with classical homogenization; however, further research work is required for reliable validation of this promising technology.

## Conflict of Interest Statement

The authors declare that the research was conducted in the absence of any commercial or financial relationships that could be construed as a potential conflict of interest.
